# A Decrease in Temperature and Humidity Precedes Human Rhinovirus Infections in a Cold Climate

**DOI:** 10.3390/v8090244

**Published:** 2016-09-02

**Authors:** Tiina M. Ikäheimo, Kari Jaakkola, Jari Jokelainen, Annika Saukkoriipi, Merja Roivainen, Raija Juvonen, Olli Vainio, Jouni J.K. Jaakkola

**Affiliations:** 1 Center for Environmental and Respiratory Health Research, University of Oulu, P.O. Box 5000, FI-90014 Oulu, Finland; kari.jaakkola@mil.fi (K.J.); jouni.jaakkola@oulu.fi (J.J.K.J.); 2 Medical Research Center, University of Oulu and Oulu University Hospital, FI-90014 Oulu, Finland; olli.vainio@oulu.fi; 3 Centre for Military Medicine, the Finnish Defence Forces, P.O. Box 5, FI-11311 Riihimäki, Finland; 4 Faculty of Medicine, University of Oulu, P.O. Box 5000, FI-90014 Oulu, Finland; jari.jokelainen@oulu.fi; 5 Unit of General Practice, Oulu University Hospital, FI-90220 Oulu, Finland; 6 Impact Assessment Unit, Department of Health Protection, National Institute for Health and Welfare, P.O. Box 310, FI-90101 Oulu, Finland; annika.saukkoriipi@thl.fi; 7 Viral Infections Unit, Department of Infectious Disease, National Institute for Health and Welfare, P.O. Box 30, FI-00271 Helsinki, Finland; merja.roivainen@outlook.com; 8 Department of Otorhinolaryngology Kainuu Central Hospital, Sotkamontie 13, FI-87140 Kajaani, Finland; raija.juvonen@kainuu.fi; 9 Northern Finland Laboratory Centre (NordLab), FI-90220 Oulu, Finland; 10 Research Unit of Biomedicine, Department of Medical Microbiology and Immunology, P.O. Box 5000, FI-90014 Oulu, Finland

**Keywords:** human rhinovirus, low temperature, absolute humidity

## Abstract

Both temperature and humidity may independently or jointly contribute to the risk of human rhinovirus (HRV) infections, either through altered survival and spread of viruses in the environment or due to changes in host susceptibility. This study examined the relationship between short-term variations in temperature and humidity and the risk of HRV infections in a subarctic climate. We conducted a case-crossover study among conscripts (*n* = 892) seeking medical attention due to respiratory symptoms during their military training and identified 147 HRV cases by real-time PCR. An average temperature, a decline in daily ambient temperature and absolute humidity (AH) during the three preceding days of the onset (hazard period) and two reference periods (a week prior and after the onset) were obtained. The average daily temperature preceding HRV infections was −9.9 ± 4.9 °C and the average AH was 2.2 ± 0.9 g/m^3^. An average (odds ratios (OR) 1.07 (95% confidence interval (CI) 1.00–1.15)) and maximal (OR 1.08 (1.01–1.17)) change in temperature increased the risk of HRV infections by 8% per 1 °C decrease. An average (OR 1.20 (CI 1.03–1.40)) and maximal decrease (OR 1.13 (CI 0.96–1.34)) in AH increased the risk of HRV infection by 13% and 20% per 0.5 g/m^3^ decrease. A higher average temperature during the three preceding days was positively associated with HRV infections (OR 1.07 (CI 1.00–1.15)). A decrease rather than low temperature and humidity per se during the preceding few days increases the risk of HRV infections in a cold climate. The information is applicable to populations residing in cold climates for appropriate personal protection and prevention of adverse health effects.

## 1. Introduction

Human rhinovirus (HRV) infections have a dominant role as causative agents in upper respiratory tract infections (RTIs) [1,2]. RTIs are the most common infections worldwide, and pose a significant public health burden in terms of absenteeism at schools and at work, increased costs related to lower work productivity, and overload of health care services. HRV infections present various clinical signs such as the common cold, otitis media, sinusitis, bronchiolitis, pneumonia, exacerbation of asthma or chronic obstructive pulmonary disease (COPD), and a HRV epidemic can eventually lead to increased mortality at the population level [2].

Cold weather-related adverse health effects are also related to behavioral, socioeconomic and housing factors which are moderated by vulnerabilities such as age, gender and comorbidities [3]. Substantial evidence exists on the seasonal variation of respiratory morbidity and mortality with the increased use of health services and hospital admissions during the winter season [4,5,6]. Environmental factors, in particular ambient air temperature and humidity, may contribute to the observed seasonal variation of respiratory viruses and the resulting RTIs [6,7,8]. All HRV species have been identified throughout the year, in temperate, tropical, subtropical, and semiarid regions [1], and peaks may occur, during autumn, winter and spring, depending on the climate and the species [2]. However, the reasons for the seasonal variation of HRV infections are not clear and their association with meteorological parameters, if any, has remained largely unknown. Experimental [7,9] and epidemiological [10,11] studies have shown that low temperature and dry air, either separately or combined, increase the risk of viral infections. Although controversial, scientific evidence suggests that inhalation of cold and dry air lowers the upper airways temperature [12,13] and dries the mucosal membrane and may cause pathophysiological responses [14] that contribute to increase the host susceptibility to viral infections. Even body surface cooling may elicit symptoms and a pathophysiological response in the host [15,16]. Also the pathogen itself may be sensitive, so that both low temperature and humidity may improve virus stability and transmission [9].

To our knowledge no previous studies examining the association between meteorological parameters and HRV infections have been conducted in cold climates involving subfreezing temperatures. Overall, only a few studies have examined the association between bio-meteorological assessments and HRV infections [8,17,18,19]. Hence, the objective of this study was to examine the relationship between short-term variations in temperature and humidity and the risk of HRV infections in a subarctic climate. To this purpose, we conducted a case-crossover study among army conscripts during their training period. Military training combines the exposure to cold and heavy physical exercises which may independently or jointly increase host susceptibility to RTIs [20,21,22]. Our hypothesis was that a decrease in daily temperature and humidity increases the risk of HRV infections.

## 2. Materials and Methods

### 2.1. Study Subjects

This work is part of a larger CIAS (Cold, Infections and ASthma) study about risk factors for asthma and respiratory infections in Finnish military conscripts [23]. Briefly, 892 men from a total of 3697 men making up two intakes of conscripts to the Kainuu Brigade in Kajaani, Northern Finland (N64°, L27°), in July 2004 and January 2005, were enrolled in this study. The subjects consisted of conscripts of whom a total of 1196 were invited to the study; 937 agreed to participate and 45 were later discharged from service because of health problems, leaving a total of 224 asthmatic and 668 non-asthmatic participants. Their mean (± SD) age was 19.1 ± 0.8 years. The enrolment procedure has been described in detail previously [23]. Each participant was followed until the end of his service lasting six to 12 months.

### 2.2. Identification of Rhinovirus Infections

Conscripts who sought medical attention for acute respiratory infections at the military primary health care clinic of the Kainuu Brigade were assessed initially by a nurse and then examined by a physician for diagnosis and treatment [23]. The day the physician was consulted for respiratory symptoms was considered the day of onset. Consultations occurring within two weeks were considered as one episode for which information of symptoms, findings and drug prescriptions were collected. The conscripts received a questionnaire inquiring about the circumstances preceding the RTI. A total of 386 sputum samples were collected and the specimens were stored at −70 °C for virological analyses. Overall, broad virus analyses (rhinovirus, influenza, adenovirus, parainfluenza, RSV, human metapneumovirus, enterovirus, *Chlamydophila pneumoniae*, and *Mycoplasma pneumoniae*) by sputum PCR and/or serology were conducted. However, the present study focuses on HRV due to scientific interest, and to the abundance of all detected viruses. HRV was identified at the sole pathogen (of those that were tested in the study) in 59 episodes and the remainder of the episodes consisted of HRV together with some other pathogen. Also other pathogens may have been present that were not tested here.

The viral RNA was isolated from 100 μL of sample with MagNA Pure LC Total Nucleic Acid isolation kit using MagNA Pure LC equipment (Roche, Mannheim, Germany) Real-time reverse transcription PCR (RT-PCR) for detection of HRV was performed [24]. The cut-off threshold was set at approximately 8% of the strongest positive control (HRV2 dilution of 10^−3^) and all plots rising above it were considered positive.

### 2.3. Exposure Assessment and Measures

Exposure assessment was based on meteorological data obtained from Kajaani Airport Meteorological Station (Paltaniemi, Finland) located at 15 km from the garrison. Average daily temperature and absolute humidity (AH) were calculated based on eight measurements conducted at three-hour intervals. A systematic review suggests that the average incubation period for HRV is 1.9 days [25] and severe symptoms peak 48 h after inoculation of the virus [26]. Therefore, an average of the three days (e.g., a 72 h-period) preceding the visit to the clinic (day 0) was calculated and used in the regression models to capture the potential effect of meteorological variables in the onset of an infection. We separately examined maximal changes, as well as the starting level from where temperature and AH changed. The meteorological conditions at day 0 were not included in the analyses.

### 2.4. Ethics Statement

The study protocol was approved by the Medical Ethics Committee of the Kainuu Central Hospital (approval No. 20/55/12.05.2003, Kajaani, Finland). The conscripts were informed of the study, possible inconvenience, confidentiality and storing of data both in written and oral form. Participation was voluntary and they could discontinue their participation at any time. The conscripts provided their written informed consent following this information.

### 2.5. Statistical Analyses

The study employed a case-crossover design which focuses on the point in time when the event occurred and is appropriate for assessing the association between short-term exposure and the risk of an acute event. For environmental temperature and AH the hazard period was defined as three days preceding the visit to the clinic for a respiratory infection. A symmetric bidirectional selection of two reference periods before and after the hazard period was utilized [27]. This means that for temperature and humidity, three-day periods in the seven days before and after the hazard period were used in the models. The statistical model compares the level and change in temperature and humidity during the days preceding the onset (hazard period) to days before and after the onset. This addresses the question of whether the onset of infection precedes unusual levels or a decline in these parameters. As the total number of episodes occurred over 85 days, meteorological information from 765 days was used in our analyses. Conditional logistic regression models were used to calculate the exposure odds ratios (OR) for the hazard compared with the reference periods. The adjusted analyses took into account the initial levels of temperature and humidity. For mean temperature and AH we adjusted for seasonal variation whereas the adjustment of the change in these parameters also considered the fact that the potential for decline in temperature and humidity depends on the starting point. Two-way interactions were tested for temperature, humidity and HRV infections. Statistical analyses were performed by SAS version 9.2 for Windows (SAS Institute, Inc.; Cary, NC, USA).

## 3. Results

**Figure 1:**
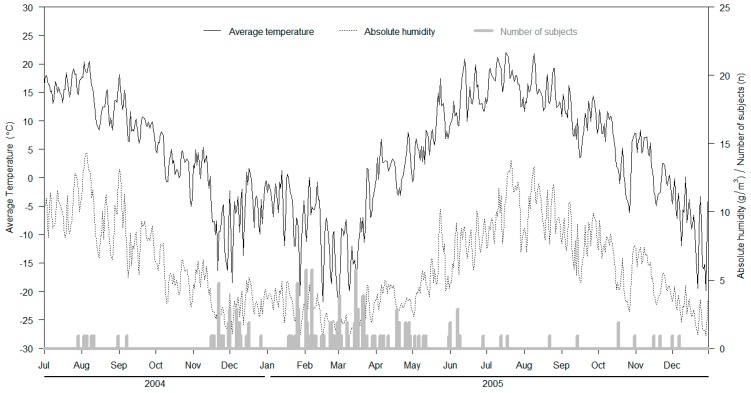
Incidence of rhinovirus infections, average temperature (°C; black solid line) and absolute humidity (AH) (g/m^3^; grey dotted line) during the study period.

Out of 386 sputum specimens, 147 (37.8%) were analysed by real-time RT-PCR [28] and found positive for HRV. A majority of the episodes were detected during the winter season and occurred as follows: 13 in November 2004, 13 in December 2004, 13 in January 2005, 29 in February 2005, 33 in March 2005, 16 in April 2005, 5 in May 2005, and 7 in June 2005. The remaining episodes were scattered over the rest of the winter season. The temperature ranged from −22.8 °C to 22.0 °C (± 10.1) and AH from 0.8 g/m^3^ to 14.3 g/m^3^ (± 3.2) during the follow-up (Figure 1). We observed that 74% of the infection episodes occurred when temperatures were ≤0 °C (109 of 147) and 26% when temperatures were above 0 °C (38 of 147). Of those conscripts infected by HRV who were engaged in outdoor training, 89% (76 of 85) reported being engaged in physical exercise, 73% (62 of 85) felt symptoms of freezing and 36% (31 of 85) reported wetting of socks or gloves during the three days prior to seeking medical consultation.

The average and mean and maximal changes during the hazard and reference periods are presented in Table 1.

We observed that the means of the meteorological conditions for the hazard period are not very different from the reference conditions.

According to the adjusted analyses, the effect estimate demonstrates an higher HRV infection risk during the preceding three days for both the mean temperature as well as its change. A reduction in the mean and maximum temperature of 1 °C increases the risk of HRV infection by 8% (Table 2). At the same time, a higher average temperature level increased the risk of HRV infection by 7%. A decrease in the mean and maximal AH during the preceding three days increased the risk by 13% and 20% for each 0.5 g/m^3^. However, despite the elevated effect estimate, the confidence intervals for mean AH change do not suggest a statistically significant effect. In addition, mean AH during the preceding three days was not significantly associated with HRV infections.

## 4. Discussion

Consistent evidence demonstrates that wintertime cold temperatures increase respiratory morbidity and mortality [4,5,11]. We conducted a case-crossover study to assess the relationship between the daily temperature and humidity and the risk of HRV infections in a subarctic climate. Our results indicate that the risk of HRV infection is associated with a decrease in both temperature and humidity during the three days preceding the infection, but that the risk in general is lower at subfreezing temperatures.

### 4.1. HRV Infections, Temperature and Humidity

Our novel finding was that a decrease in either temperature or humidity, rather than their levels per se, increases the risk of HRV infections in the three days before seeking medical care. To our knowledge this is the first study assessing relationship between short-term variation in meteorological factors and HRV infections. Our finding is consistent with the observed higher risk of influenza [11] in the same population, assessed in a smaller sample. Support for the importance of variation in temperature as a risk factor for RTIs was also recently obtained from a population study in China where large diurnal differences in temperature increase emergency visits for RTIs [19].

The reason why a decrease in temperature or AH may increase the risk of HRV infections could be related to changes in the host susceptibility. Our hypothesis was that a combination of low temperature and humidity would lead to the cooling and drying of the respiratory tract which may be enhanced during the outdoor physical activity [29] related to military training. This was supported by the cold stress reported by most of the infected individuals during the previous three days. An experimental study showed that breathing cold air during exercise reduces the upper respiratory tract temperature by several degrees and even more because of increased ventilation [13]. A sudden decrease in temperature and humidity could be related to altered airways function, increasing the susceptibility to viral agents. For example, inhaling larger volumes of air with low AH while exercising dries the mucosal membrane [12]. This can even lead to epithelial damage, depress ciliary movement in the respiratory tract [13] and increase the susceptibility to infections. Even cooling of the body surface could elicit a reflex of vasoconstriction in the nose and upper airways, inhibit the respiratory defense and convert an asymptomatic subclinical viral infection into a symptomatic clinical infection [15]. An experimental study on humans showed that acute chilling of the feet elicits symptoms of the common cold [16]. However, another study did not demonstrate altered host resistance to HRV infections as a result of whole-body cold exposure [30]. Finally, both cold exposure and heavy exercise involve stress. Stress itself may predispose the host to acute RTIs [31] and one possible reason is through the suppressed immune processes of the host [21,22]. However, it should be noted that the association between immune function and cold exposure has not been clearly demonstrated [32].

According to our analyses HRV infections were positively related to the mean temperature. This seemingly contradictory finding could be related to the viability and transmission of the pathogen. It is possible that at very low temperatures, the transmission and survival of the virus decrease, as opposed to conditions where temperatures approach zero degrees, and where we observed most of the respiratory tract infections [10]. Previous experimental research on influenza in animal models has suggested that both low temperature and AH increase the viability and transmission of influenza. Low temperature itself could improve viral stability, increase shedding and affect the physical properties of the pathogen [9]. On the other hand, for some viruses, such as respiratory syncytial virus (RSV), elevated temperature may induce conformational changes and increased fusion activity [33]. HRV replication has been shown to improve when the temperature resembles the one in the upper (33 °C) compared to the lower (35 °C) airways [34]. Although we did not observe an association between mean AH and HRV infections, previous experimental studies have shown that both airborne and contact survival of HRV decrease when the relative humidity (RH) ranges from low to moderate (20%–50%) but improves when the RH is high (80%) [35,36]. Even a bimodal pattern for some pathogens has been suggested with altered virus survival and transmission at very low to high humidity [37]. Low humidity could favor viability and transmission of viruses within aerosols [9] which could be an important mode of transmission in cold and dry climates [7,9,37]. The primary route of HRV transmission is controversial and may vary according to environmental conditions either as airborne or contact transmission [1,2]. In addition, our study design does not enable assessment of the specific environmental or host conditions (e.g., role of co-infections) that occurred at the time of the acquisition, or shedding of the virus. It should be noted from our study that in addition to low wintertime outdoor temperature, dry air affects residents both indoors and outdoors.

### 4.2. Seasonality of HRV Infections

The unique aspect of our study is related to the considerable annual variation in temperature and humidity. We demonstrated that although HRV occurred throughout the year, distinct peaks were observed during the winter season and early spring. The observed HRV infections appeared before, during and after the detected influenza episodes in this study [11] and were not related to the enrolment in military service which occurs twice a year, in July and January. A previous report from the present data showed that all known HRV types were present in the sputum of conscripts: HRV-A strains were discovered steadily throughout the year while HRV-C strains were present in winter and spring specimens and HRV-B strains were found scarcely and mainly during the summer months [28].

There are relatively few studies reporting the association between HRV infections and meteorological parameters. A study investigating hospitalized children from Germany over a five-year period detected that HRV infections occurred more often when the mean (two weeks’) relative humidity was higher. In contrast, low temperature was associated with higher hospitalizations for other acute respiratory illness pathogens [8]. Gardinazzi [18], on the other hand, studied monthly temperature and humidity related with children hospitalized with viral respiratory infections in Brazil, and observed that the infections occurred during the coldest and driest months. Similarly, a recent study from Africa detected that respiratory infection epidemics occurred when mean monthly night-time temperatures and humidity were at their lowest [17]. A comparison between the different studies is difficult or impossible due to variations in climate (mode of transmission), as well as different approaches to study the association between exposure (e.g., seasonal, short-term) and outcomes.

Overall, the reasons for the seasonality of HRV pathogens can be related to the previously mentioned effects of climatic factors on the survival and spread of viruses in the environment; due to changes in the population’s susceptibility to acute RTIs (e.g., seasonal changes in the immune function), and related to the physiological reactions of the host to certain climatic conditions (e.g., breathing cold and dry air) or due to altered transmission of pathogens responsible for acute respiratory illnesses through changing host behavior (e.g., crowding) in certain climatic conditions [6]. The significance of meteorological parameters as determinants of the seasonal variation of respiratory infections is being debated. The concern is timely due to the expected increased variability in winter weather patterns as a result of the climate change.

### 4.3. Strengths and Limitations of the Study

We used a case-crossover design where time-invariant confounders are controlled by making within-subject comparisons, and time-varying confounders (e.g. seasonal variation in determinants of HRV) by matching referents to the index time [27,38]. Compared to the general population, conscripts are more frequently exposed to cold and physical exercise, which provides additional strength to this study. Military service is mandatory for young men in Finland, and conscripts represent the ordinary and healthy population of this group of age. Despite the case-crossover design, the effect of crowding cannot be excluded. Previous research has shown that that military training is related to a broad spectrum of respiratory pathogens [39] that are effectively spread [21] and can cause mild or severe outbreaks of acute respiratory diseases [20,40]. However, in our study no clear effects related to the onset of military service and the occurrence of HRV infections were observed. In addition, crowding remained constant throughout the follow-up and has not likely affected the main results of our study. One possible limitation is that seeking of medical care may be delayed due to individual differences in incubation times and responses to cold exposure, as well as related to symptoms of HRV infections sometimes being mild. However, on the other hand, we assume that the outdoor training reported frequently in this study, including heavy exercise and chilling and preceding the onset of infections could have worsened the symptoms and favored care-seeking. Another limitation is that the specimens of this study were sputa collected at times of any RTI and it cannot be ruled out that they may have been contaminated with pharyngeal HRV.

## 5. Conclusions

Our results show that a decrease in temperature and AH is associated with the increased occurrence of HRV infections. In contrast, the risk of HRV infections is reduced at subfreezing temperatures and higher AH. The information is applicable to all populations residing in moderate or cold climates. Individuals and healthcare professionals need to be aware of lowered temperature and humidity as contributing factors in the development of HRV infections. Appropriate whole-body and respiratory protection may reduce potential cold-related adverse respiratory health effects.

## Figures and Tables

**Table 1:** Mean and maximal decline in temperature and absolute humidity (AH) during the hazard period (*n* = 146) prior to the onset of rhinovirus infections and during the reference periods. Values represent mean ± standard deviation (SD).

Parameter	Hazard	Reference ^1^ (Pre)	Reference ^1^ (Post)
**Temperature (°C)**			
mean	−9.9 ± 4.9	−9.7 ± 4.5	−8.1 ± 4.8
mean change	1.5 ± 3.1	−0.3 ± 4.6	1.9 ± 3.9
maximum change	4.1 ± 3.1	2.4 ± 4.4	6.0 ± 5.1
**Absolute humidity (AH)**			
mean	2.2 ± 0.9	2.3 ± 0.9	2.4 ± 0.8
mean change	0.2 ± 0.6	−0.1 ± 0.8	0.2 ± 0.6
maximum change	0.7 ± 0.6	0.4 ± 0.7	0.6 ± 0.7

^1^ indicates a reference period of three days either seven days prior to or after the hazard period.

**Table 2:** Onset of a human rhinovirus (HRV) infection (*n* = 146) and its association with mean values and declines in temperature (per 1 °C) and humidity (0.5 g/m^3^).

Parameter	OR (95% CI) ^1^	Adjusted OR (95% CI) ^2^
**Absolute humidity (AH)**		
mean of three prior days	0.94 (0.86–1.03)	0.97 (0.80–1.16)
maximum change during three prior days	1.09 (0.96–1.24)	1.20 (1.03–1.40)
mean change during three prior days	1.05 (0.91–1.21)	1.13 (0.96–1.34)
**Temperature (°C)**		
mean of three prior days	0.96 (0.92–1.00)	1.07 (1.00–1.15)
maximum change during three prior days	1.04 (0.98–1.10)	1.08 (1.01–1.17)
mean change during three prior days	1.04 (0.97–1.11)	1.08 (1.01–1.17)

^1^ The odds ratios (OR, 95% confidence interval) were calculated per 1 °C temperature and per 0.5 g/m^3^ absolute humidity decreases; ^2^ Adjusted for the initial level of the temperature and AH. The adjusted mean temperature and AH take into account seasonal variation, whereas the adjusted change in these parameters also considers that the potential for change in temperature and humidity depends on the level of the parameters. CI: confidence interval.
